# Supplementary material for: Splice-Junction-Based Mapping of Alternative Isoforms in the Human Proteome
Source: Cell Rep. Author manuscript; Available in PMC 2020 Jan 15. (PMC6961840; doi:10.1016/j.celrep.2019.11.026)
Supplement: 2 [file NIHMS1546469-supplement-2.zip › DF1/PXD006675/Aorta/Aorta_18_SCAMP2_TASGSPAISGTNPADPPGSADK.pdf]

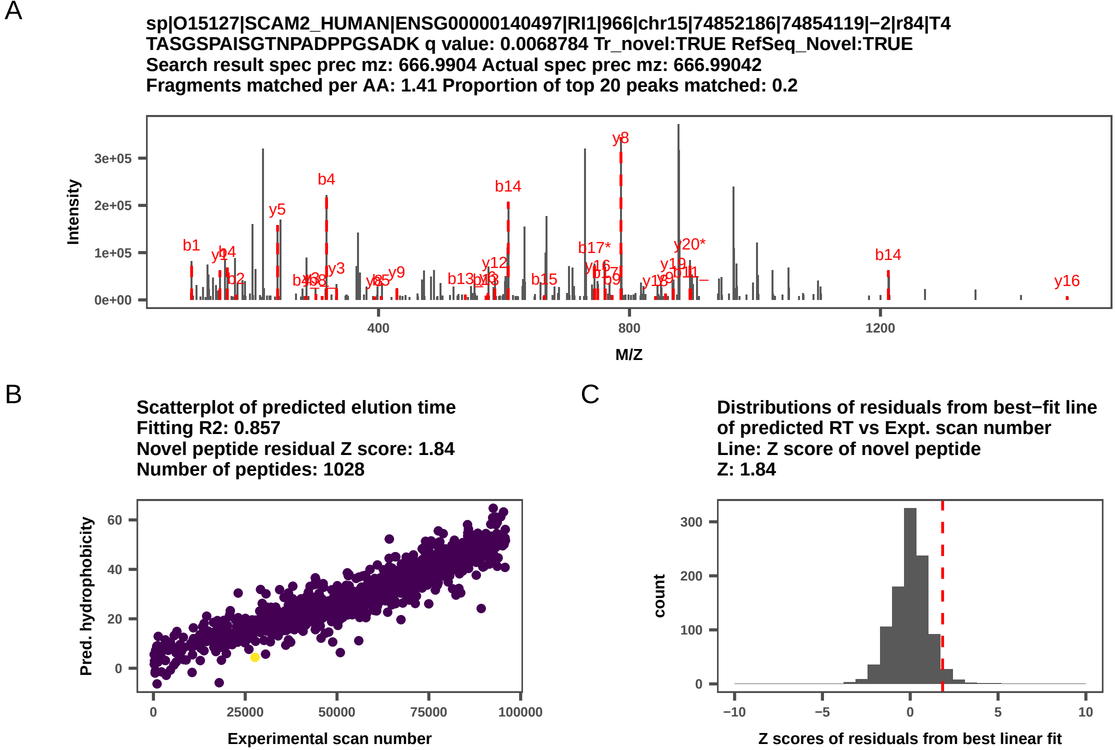
A
sp|O15127|SCAM2_HUMAN|ENSG00000140497|RI1|966|chr15|74852186|74854119|−2|r84|T4
TASGSPAISGTNPADPPGSADK q value: 0.0068784 Tr_novel:TRUE RefSeq_Novel:TRUE
Search result spec prec mz: 666.9904 Actual spec prec mz: 666.99042
Fragments matched per AA: 1.41 Proportion of top 20 peaks matched: 0.2
Intensity
3e+05
2e+05
1e+05
0e+00
M/Z
400
800
1200
y8
b4
b14
y5
b1
b17*
y20*
b14
y12
y16
y9
y3
b2
b13
b15
y19
y18
B
Scatterplot of predicted elution time
Fitting R2: 0.857
Novel peptide residual Z score: 1.84
Number of peptides: 1028
Pred. hydrophobicity
60
40
20
0
0
25000
50000
75000
100000
Experimental scan number
C
Distributions of residuals from best−fit line
of predicted RT vs Expt. scan number
Line: Z score of novel peptide
Z: 1.84
count
300
200
100
0
−10
−5
0
5
10
Z scores of residuals from best linear fit
